# Optimal Timing of Anterior Cruciate Ligament Reconstruction: A Narrative Review of the Intra-articular Consequences of Prolonged Injury-to-Surgery Intervals

**DOI:** 10.7759/cureus.95620

**Published:** 2025-10-28

**Authors:** Raed Y Abudaqqa, Ali J Al Mas, Faris A Abushaaban, Kariyal P Arun, Ahmad Rebhi Abed

**Affiliations:** 1 Alkhor Hospital, Hamad Medical Corporation, Doha, QAT; 2 Orthopedic Surgery, Board of Orthopedic Surgery, Hamad Medical Corporation, Doha, QAT

**Keywords:** anterior cruciate ligament reconstruction (aclr), chondral defect, complex meniscal injury, injury-to-surgery delay, knee instability, medial meniscus, meniscal tear, surgical timing

## Abstract

This study aims to determine how the timing of anterior cruciate ligament (ACL) reconstruction affects the prevalence and severity of associated meniscal and chondral injuries. This literature review critically synthesizes existing research on the timing of ACL reconstruction and the impact of prolonged injury-to-surgery intervals on intra-articular knee lesions and functional outcomes. It examines evidence concerning the correlation between surgical timing and intra-articular knee injuries that supports or refutes the hypothesis that longer intervals from injury to surgery worsen intra-articular pathology. This correlation is essential to guide surgical decision-making, optimize treatment timelines, and avoid long-term joint degeneration. Ultimately, delays beyond six months significantly increase the likelihood of irreparable meniscal tears and poorer functional outcomes.

## Introduction and background

Injuries to the anterior cruciate ligament (ACL) are among the most common ligamentous injuries affecting the knee joint and are frequently seen in active and young populations who engage in sports or high-demand physical activity [1]. The ACL is a critical stabilizer of the knee, resisting anterior tibial translation and rotational forces [2], and rupture of the ACL compromises both mechanical and proprioceptive stability. Isolated ACL tears are common, but delayed reconstruction results in secondary injuries to intra-articular structures, most commonly the menisci and articular surfaces [3].

A wide variation in the incidence of meniscal injury in ACL-deficient knees has been reported, ranging from 40% to over 80%, and the incidence increases with longer intervals from injury to surgical intervention [4,5]. In particular, medial meniscus tears (MMTs) are more common with chronic ACL deficiency, when the medial meniscus acts as a secondary stabilizer in the absence of an intact ACL [6]. Conversely, lateral meniscus tears (LMTs) frequently occur acutely with the injury, often through a pivot-shift mechanism [7]. Several studies have reported that delayed ACL reconstruction considerably increases the risk of medial meniscus damage, especially after 6 months post-injury [8,9].

Chondral defects are also increasingly recognized as a serious consequence of untreated ACL injury. Repetitive microtrauma and abnormal joint loading that occur during instability episodes of the ACL-deficient knee contribute to these defects [10]. The grades of chondral injuries are often indexed using the Outerbridge classification, ranging from Grade I (softening) to Grade IV (exposed subchondral bone) [11]. Progression from Grade I-II to Grade III-IV is time-dependent and closely related to instability duration [12]. Both clinical and arthroscopic evidence support that delayed ACL surgery, usually beyond 6-12 months, increases the incidence and severity of chondral lesions [9].

The biomechanics of an ACL-deficient knee alter kinematic patterns, increasing anterior translation and internal rotation during movement and generating abnormal shear forces across the meniscus and articular surfaces [13]. These forces lead to progressive degeneration, most notably of the weight-bearing aspects of the medial femoral condyle and tibial plateau [14]. The cumulative trauma to intra-articular structures is worsened by repeated episodes of giving way or subluxation, underscoring the importance of timely surgical management [15].

The focus of this study is to determine how the timing of ACL reconstruction affects the prevalence and severity of associated meniscal and chondral injuries. This review analyzes previously published research on the timing of ACL reconstruction to provide objective evidence that supports or refutes the hypothesis that prolonged intervals from injury to surgery worsen intra-articular pathology. This correlation is essential to guide surgical decision-making, optimize treatment timelines, and avoid long-term joint degeneration.

## Review

Methodology

This literature review aims to analyze and synthesize published articles on the optimal timing for ACL reconstruction (ACLR) and the impact of a prolonged injury-to-surgery interval on intra-articular knee consequences. With a rigorous and transparent approach, the review was guided by the following keywords: ACLR, meniscus tear, ACL reconstruction, meniscal repair, injury-to-surgery time, delayed ACL reconstruction, chondral lesions, osteoarthritis, functional knee outcome, chronic ACL timing, intra-articular consequences, and arthroscopy. A systematic search was conducted across multiple electronic databases like PubMed, MEDLINE, Scopus, Cochrane Library, and Google Scholar to identify all relevant articles that were published in the English language and met the inclusion criteria. The inclusion criteria were primary ACL reconstruction, studies investigating the timing of ACLR, studies on injury-to-surgery intervals of ACLR, and studies examining the relationship between the timing of ACLR and meniscal and intra-articular knee consequences. On the other hand, revision cases, multiple-ligament knee reconstruction cases, and non-English articles were excluded.

Moreover, a variety of methodological approaches were reviewed, predominantly ranging from retrospective cohort studies to systematic reviews and meta-analyses, with some prospective randomized controlled trials. These reviews frequently highlight methodological inconsistencies across included studies, such as subjective-objective outcome measures (e.g., Lysholm, IKDC), patient geographic variables, graft types, postoperative MRI, and rehabilitation protocols, regarding delay intervals for ACL reconstruction.

Literature review

According to current orthopedic guidelines, early intervention in ACL injuries, particularly in patients demanding high activity or those with instability episodes, is recommended [16]. Reconstruction early has been associated with lower rates of secondary injuries [17] and better long-term functional outcomes, as well as a smaller risk of developing osteoarthritis. Yet, the optimal timing of surgery remains controversial, in part because different studies use varying definitions of “early” and because patient factors may influence postoperative timing [18].

Much recent attention has been focused on the timing of ACLR, with associated intra-articular injuries such as meniscal and chondral injuries being in focus. A variety of studies suggest that an interval extending over a protracted period of time between injury and surgery may have a major impact on the incidence and severity of such concomitant injuries, commonly with attendant adverse effects on the health and functioning of the knee joint [19].

Compression of both the medial and lateral menisci preserves load while reducing it in the unaffected compartment, supports joint stability, and helps prevent cartilage degeneration. Chronic ACL deficiency increases mechanical strain on the menisci, particularly the medial meniscus, which replaces the lack of stability provided by the ruptured ACL [20]. Boks et al. found that medial meniscus tears were more frequent in patients who underwent delayed ACL reconstruction as opposed to patients who had ACL reconstruction more rapidly, indicating that it is the time to surgery that plays a predominant role in meniscal preservation [21]. Ahlen et al. also observed a much higher rate of meniscus resection in patients whose surgery was delayed more than 12 months post-injury [22].

Unlike lateral meniscus injuries, which tend to occur more acutely at the moment of injury, medial meniscus injuries often occur as a result of repetitive overloading of the joint. Many researchers have shown that the lateral meniscus tear is the most common tear noted in early surgical cases and the first traumatic lesion to be associated with the pivot shift mechanism of ACL injuries [23]. Nevertheless, these acute lateral tears may transform into chronic, irreparable forms with delayed reconstruction, and the result is more extensive resection and more cartilage wear with time [24].

Similarly, the adverse effect of delayed surgery on osteochondral injury has also been addressed well. Repetitive shear and compressive forces secondary to ongoing instability result in extreme vulnerability to articular cartilage. Cartilage lesions in ACL-deficient knees were studied by van der Hart et al., who found that the prevalence of chondral defects increased significantly with the duration of an ACL-deficient state and that the most common location was the medial femoral condyle [25]. In a separate study, Tandogan et al. reported that Grade III and IV cartilage lesions were predominantly seen in patients where surgery was delayed beyond six months and that delayed instability accelerates degenerative processes [26].

Additional studies have tried to determine how the time to surgery affects the progression of intra-articular damage. In a prospective analysis of over 1200 patients, Wasserstein et al. showed a linear increase of about 5% per one month delayed from injury to surgery in the risk of developing meniscal and chondral injuries, which also supports early surgical management [27]. Fithian et al., another extensive cohort analysis, demonstrated that confining surgery to 12 weeks from injury dramatically decreased the risk of delayed development of irreparable medial tears and subsequently affected the postoperative functional outcomes [28].

Also, the extent of secondary injury beyond the knee injury may depend on demographic and injury-specific variables in ACL-deficient knees. Recurrent instability episodes are particularly dangerous for younger, more active patients because there is an increased chance of further damage. On the other hand, sedentary patients can endure less mechanical stress with rarely maintained intra-articular structure, and delayed surgery may proceed with little drawback [29]. The observation of this is not surprising and emphasizes the need for personalized determination of surgical timing based on patient activity level, age, and need for function [30].

Progressive tissue degeneration within the knee joint following ACL injury is also associated with biological changes within the knee joint. It has been shown that proinflammatory cytokines secreted as a result of ligament rupture can generate a catabolic environment within the joint, which permits collagenase degradation and inhibits healing [31]. Spindler et al. and Bedi et al. studied elevated levels of matrix metalloproteinases (MMPs) and interleukin-1 beta (IL-1β) in ACL-deficient knees, which correlated with cartilage softening and early osteoarthritis [32].

In addition, patients who had surgery within three months of injury were significantly more likely to undergo meniscal repair, rather than resection, and achieved better long-term outcomes, therefore suggesting patients should have surgery as soon after injury as possible [33]. On the other hand, chronic cases often need partial or complete meniscectomy, whose association with the onset of early osteoarthritis and inferior knee functioning over time is well known [34].

The cumulative data also suggest that the long-term outcome of ACL reconstruction is dependent upon the condition of the knee joint at the time of surgery. Magnussen et al. showed in a large multicenter study that ACL reconstruction in the presence of cartilage lesions or untreated meniscal pathology was predictive of poorer patient-reported outcomes and more rapid radiographic appearance of osteoarthritis [35]. These findings demonstrate the importance of surgical timing to retain joint health and maximize natural recovery.

Lastly, surgical outcomes and rehabilitation protocols are influenced by the type and grade of osteochondral lesions in ACL-deficient knees. However, Outerbridge grading continues to remain the most popular system of grading osteochondral damage, and Grade III-IV lesions represent the appropriate lesions with significant cartilage damage. Gillquist and Messner’s research showed that knees with higher-grade OCD lesions at the time of reconstruction developed a more delayed return to activity and post-reconstruction stiffness compared to those with minimal chondral changes [36]. Early detection and early surgery may prevent the irreversible intra-articular deterioration we see with the interval between presentation and surgery.

Discussion

According to the results of this literature review, delayed ACLR has important clinical implications for intra-articular structures, specifically the menisci and chondral surface. Most of the reviewed studies show that the rate of medial meniscus injury and chondral injury rises steadily with time from injury to surgery of ACLR. These findings support the thesis that chronic knee instability in ACL-deficient knees accumulates biomechanical stress to the medial knee compartment, leading to irreversible progressive chondral cartilage damage.

The role of the medial meniscus in stabilizing the ACL-deficient knee has been highlighted in numerous biomedical studies. In a deficient ACL knee joint, anterior tibial translation is prevented by the medial meniscus, particularly under rotational stress [36].

Clinical evidence indicates that this prolonged compensatory load contributes to chronic microtrauma and degenerative changes, a condition confirmed by cadaveric and in vivo models [37]. Most of the reviewed studies support this thesis by showing a significant correlation between the medial meniscus and chondral injuries and prolonged injury-to-surgery intervals when ACLR was performed more than six months after injury.

Additionally, according to Wasserstein et al., patients who underwent ACL reconstruction at or after 12 months had significantly higher odds of irreparable meniscal injury compared with those operated on earlier. Furthermore, repairing complex meniscus tears is more difficult and is associated with inferior functional outcomes and an increased risk of progression of degenerative changes in the knee joint. Likewise, patient demographic factors such as BMI, gender, age, and mechanism of injury can impact concomitant knee injuries like meniscal and chondral damage. Previous studies have shown higher BMI as a risk factor for primary ACL injury, recurrent instability, and complications [38-41].

The mechanism of injury also remains an important risk factor for meniscal and chondral injury. The majority of ACL injuries occur due to sport-related activities such as football, basketball, and other contact sports, which are known for their tremendous torsional and valgus forces that result in ligamentous injury and meniscal entrapment or detachment [41,42]. Consistent with the literature, high-energy twisting mechanisms are associated with increased intra-articular knee joint damage present at the time of initial injury [43]. This review reinforces the need for a detailed injury history during preoperative planning in order to establish a statistical association between the mechanism of injury and meniscal pathology.

The results of this review strongly endorse the idea of early ACL reconstruction, ideally within the first three to six months post-injury, from a surgical planning perspective. Despite the ongoing debate about the necessity of early intervention for all patients, particularly those with low activity levels, the relationship between surgical delay and complex intra-articular pathology reinforces the argument for early operative management in young, active individuals [9].

Postponing ACL reconstruction significantly increases the likelihood of complex, irreparable meniscal tears that could adversely affect long-term functional outcomes. The reviewed articles indicate that minimizing the injury-to-surgery interval reduces the risk of knee joint damage.

There are several limitations of this literature review. Firstly, it is constrained by the availability of published articles, which limits the depth of analysis. Secondly, the selection process can introduce researcher bias. Lastly, a literature review is primarily synthesized from existing published knowledge.

## Conclusions

This literature review underscores that prolonged injury-to-surgery time of ACLR is associated with a higher risk of complex meniscal injuries and progressive chondral degeneration. Delays beyond six months significantly increase the likelihood of irreparable meniscal tears and poorer functional outcomes. Overall, the evidence supports early ACLR, ideally within three to six months, to minimize secondary joint damage and optimize outcomes.
